# A Psychometric Measure of Working Memory Capacity for Configured Body Movement

**DOI:** 10.1371/journal.pone.0084834

**Published:** 2014-01-21

**Authors:** Ying Choon Wu, Seana Coulson

**Affiliations:** 1 Center for Research in Language, University of California San Diego, La Jolla, California, United States of America; 2 Swartz Center for Computational Neuroscience, University of California San Diego, La Jolla, California, United States of America; 3 Cognitive Science Department, University of California San Diego, La Jolla, California, United States of America; Emory University, United States of America

## Abstract

Working memory (WM) models have traditionally assumed at least two domain-specific storage systems for verbal and visuo-spatial information. We review data that suggest the existence of an additional slave system devoted to the temporary storage of body movements, and present a novel instrument for its assessment: the movement span task. The movement span task assesses individuals' ability to remember and reproduce meaningless configurations of the body. During the encoding phase of a trial, participants watch short videos of meaningless movements presented in sets varying in size from one to five items. Immediately after encoding, they are prompted to reenact as many items as possible. The movement span task was administered to 90 participants along with standard tests of verbal WM, visuo-spatial WM, and a gesture classification test in which participants judged whether a speaker's gestures were congruent or incongruent with his accompanying speech. Performance on the gesture classification task was not related to standard measures of verbal or visuo-spatial working memory capacity, but was predicted by scores on the movement span task. Results suggest the movement span task can serve as an assessment of individual differences in WM capacity for body-centric information.

## Introduction

The concept of working memory (WM) was developed to explain how limited amounts of information can be maintained and manipulated in active consciousness for short periods of time. In the now classic model advanced by Baddeley and Hitch [1], WM is comprised of multiple components, including the so-called central executive and at least two slave systems for the maintenance and manipulation of modality-specific information. The central executive regulates attentional resources and controls the flow of information, while distinct, modality-specific sub-systems maintain what is selected for processing. Visual and spatial information is mediated by the visuo-spatial sketchpad (VSSP), and acoustic or verbal information, by the phonological loop. An additional component – the episodic buffer – is proposed to mediate the integration of information from a variety of sources, including other WM subsystems and long term memory, leading to complex representations, such as episodes [2], [3].

The present study concerns immediate memory for body configurations and movements. On the basis of the multi-component WM model outlined above, we might expect memory for body movements to tap visuo-spatial resources, particularly in the case of learning a novel movement sequence through observation. However, dual task research has revealed a double dissociation between memory for spatial locations and memory for body movements, suggesting the classic WM model should be augmented to include a sub-system for the maintenance of memory for body postures [4]. For example, when the secondary task involves tapping spatial locations in sequence, little or no interference in remembering meaningless body movements has been reported relative to a baseline with no secondary task [4]. In contrast, performance is substantially impacted when the secondary task taps motor resources, as in squeezing a tube [5], and copying, or even simply watching, body movements [4], [6], [7].

The reciprocal is also true. Spatial tapping or other target-directed movements have been shown to interfere with primary tasks that involve remembering locations in space, such as the Corsi block task [7], [8], the Brooks matrix task [6], and others [9], whereas the production of patterned body movements does not.

These studies are complemented by work suggesting that aspects of configured body movement, such as the serial order of target presentation, are stored separately from spatial location of targets [10]. Additionally, oscillatory EEG activities above 13 Hz have been shown to respond differently to WM tasks involving the retention of spatial targets that guide movement versus those that involve a change detection task [11]. These findings are consistent with the notion of distinct processing streams in WM for visual versus kinesthetic aspects of space.

The notion that remembering body postures and spatial locations may be mediated by only partially or non-overlapping cognitive systems is particularly germane to the growing literature that highlights sensori-motor contributions to WM [12], [13]. Differential patterns of cortical activation during the rehearsal of visually presented objects that were either manipulable (e.g. a key) or non-manipulable (e.g. a sun) have led researchers to propose that manual action representations may contribute to the maintenance of graspable objects in temporary memory stores [14]. Together with the dual task research, studies such as these raise the possibility that body-specific representations or processes might contribute to WM function independently of verbal or visuo-spatial modalities.

To explore this possibility, we created an assessment tool dubbed the movement span task. In this task, participants view sets of short video clips of a person producing between one and five meaningless movements. After viewing each set, the participant reproduces the target movements. The goal of the present study was, first, to establish the extent to which participants' performance on the movement span task was correlated with their performance on span tasks assessing verbal and visuo-spatial working memory, and, second, to test whether performance on the movement span task was predictive of success on a novel task involving body movements accompanying conversational speech (namely, co-speech gestures).

The movement span test was modeled after existing measures designed to probe visuo-spatial or verbal WM capacity, such as the sentence span task and the Corsi block task. The hallmark of these assessments is that participants are required to actively maintain increasing loads of information as they perform a concurrent processing task in the same domain. For example, in the sentence span task, participants must remember sets of two, three, four, or five words in the face of interference from a secondary task that involves listening to and comprehending a series of spoken, unrelated sentences. In the case of the Corsi block task, participants view an asymmetric grid of blocks that the experimenter taps in sequences ranging from four to nine items. The participant is then requested to tap the same blocks in the order that they were touched by the experimenter. Although the classic version of this task does not involve a secondary interference task, the Corsi block task is generally thought to recruit central executive resources due to the requirement for sequential recall [15].

An individual's span score is typically operationalized as the highest level at which a predetermined quantity of trials or items can be correctly recalled [16]. For instance, the Corsi block span is the highest level at which an entire sequence of block locations is reproduced. On the other hand, because the listening span task does not require sequential recall, listening span scores reflect the highest level at which at least a subset of the total items presented are recalled. By analogy, since the movement span task also involves free recall, an individual's absolute span score is defined as the level at which at least half of all items were accurately recalled.

Because pilot testing revealed that the task of reproducing a series of meaningless body movements was itself quite taxing, additional cognitive loads in the form of a secondary interference task or serial recall procedure were not used. Notably, each item in the task required the coordination of at least two effectors along a variety of dimensions, including location, orientation, and hand shape. Additionally, most items involved a sequential combination of elemental movements (e.g., extending the left arm and then sliding the right hand from the left wrist to the left shoulder). For these reasons, it is likely that remembering the stimuli presented in the movement span task tapped central executive resources in a manner analogous to the serial recall procedure used in the Corsi block task.

If immediate memory for body postures and movements is – at least to some degree – independent of other modality specific subsystems of WM, we would expect individual differences in this capacity to uniquely predict performance on tasks that depend heavily on body movement processing. An example of such a task is the comprehension of co-speech gestures, which are body movements that speakers produce in the course of everyday conversation. Such gestures can serve a number of functions, including guiding attention [17], [18], facilitating interaction [19], or depicting visuo-spatial aspects of discourse referents [20], [21], [22].

Numerous studies indicate that listeners are sensitive to the gestures that accompany their interlocutors' speech (for reviews, see Goldin-Meadow [23], [24]). Further, listeners are able to combine information made available in these two channels [22], [25], [26], [27], [28]. Because the spoken and gestural portions of a speaker's utterance unfold dynamically along complementary time courses, it is likely that listeners recruit WM as they interpret discourse accompanied by gestures. Further, because co-speech gestures invariably involve the body, speech-gesture integration is precisely the type of process that we would expect to recruit a body-specific WM subsystem.

Accordingly, we created a gesture classification task intended to assess participants' sensitivity to co-speech gestures. In this task, participants viewed several short, video-recorded segments of spontaneous discourse. In half the trials, audio speech tracks were presented with the original iconic gestures that had accompanied them. In the other half, the speech and gesture portions of the videos were swapped such that a semantic relation between the two was difficult to apprehend. Participants were asked to categorize the gestures in each trial as either congruent or incongruent with the speaker's speech.

In the present study, we administered the movement span, sentence span, and Corsi block tasks, along with the gesture classification task to 90 healthy adults, and tested for a predictive relationship between span scores and d' obtained from each participant's accuracy on the gesture classification task. If movement span is a valid assessment of a body specific WM sub-system, we would expect it to predict signal detection on the gesture classification task. Alternatively, success on the gesture classification task might be predicted by traditional measures of visuo-spatial or verbal WM capacity.

## Methods

### Participants

90 healthy adults from the UCSD community (52 female) received academic course credit for participating in the battery of tests. All volunteers were fluent in English and gave written informed consent. This study was approved by the Human Research Protections Program of UC San Diego.

### Materials, Design, and Procedure

The study was comprised of the movement span task, the sentence span task, the Corsi block task, and the gesture classification task. Before each test, the experimenter gave instructions both verbally and in written form. Next, participants completed a practice block and were given the opportunity to ask questions. In the case of the movement span task, participants also gave consent to be videotaped. This test took place in a larger room with the experimenter continually present. A Flip video camera was used for filming, and digital videos were used later for off-line scoring. The remaining portions of the experiment (the sentence span task, the Corsi block task, and the gesture classification task) were completed in private booths, each equipped with a PC, a flat screen monitor, and speakers. Data acquisition and stimulus presentation were done with DataRiver [29] for the movement span, Corsi block, and gesture classification tasks, and E-Prime software for the sentence span task.

Movement Span Task. corpus of over 100 meaningless movements was constructed by first establishing twenty basic meaningless configurations. Care was taken to include a variety of axes of rotation (e.g., wrist, elbow, shoulder, fingers). Families of movements were then derived by modulating relevant parameters, such as orientation, trajectory, and hand morphology. An actress was videotaped enacting each item. From the continuous video, 77 items were extracted that ranged in length from 2.1 to 2.4 seconds.

To ensure that these meaningless movements were indeed uninterpretable, twelve volunteers (who did not participate in any other portion of this study) were presented with the 77 meaningless experimental stimuli along with 60 control videos in which the same actress pantomimed meaningful actions (coughing, yawning, nodding, and so forth). Participants in the norming study were instructed to select the word that best described what the person was doing in each video from three possible choices. *Meaningless* was a fourth option, to be chosen if the participants were unsure. 73 of the original 77 meaningless trials were accurately classified as such by at least 91% of participants.

Of the accurately classified items, 45 were selected for inclusion in the movement span task (see http://bclab.ucsd.edu/movementSpanMaterials for videos, as well as other materials and documentation). Care was taken to select items that involved a wide variety of hand/arm configurations and movement trajectories in order to minimize proactive interference. The majority of movements were asymmetric (85%). Also in the majority of items (80%), arms, elbows, and shoulders contributed to the primary defining dimensions of the movement, while both hands were held in either a flat hand shape – akin to the ASL sign for the letter B – or a fist. The remaining targets were derived principally through hand and finger configurations. Roughly equal proportions of items involved either a trajectory along a vertical axis extending from waist level to above the head, or they were executed in a central space in front of the chest, neck, or chin. Ten additional stimuli were performed either along a horizontal or front-back axis at mid torso or waist level. Please consult supplementary materials (http://bclab.ucsd.edu/movementSpanMaterials) for a catalogue outlining relevant movement parameters, such as main effectors and principle axes of rotation, as well as descriptions of each item.

Previous research suggests that when given the opportunity to practice all target movements beforehand, healthy adults are typically able to accurately reproduce up to four meaningless movements at a time on average [7], [8] Because in the present paradigm, each item was novel to the participant at the time of encoding, the average movement span was expected to be smaller – and for this reason, five levels were included in the task. The range of possible absolute movement span scores (one through five, with five levels total) is comparable to the scoring parameters of both the sentence span (five levels) and Corsi block (six levels – although the Corsi block task contains nine levels total, participants start out on level four because healthy adults typically perform at ceiling when the memory load is less than four).

On the first level, participants viewed one movement on a computer screen, immediately followed by an auditory cue to begin reproducing what they had seen with their own hands and arms. Once finished, they clicked a mouse to advance to the next trial. On the second level, two movements were presented in succession, followed by the recall cue; on the third level, three movements, and so forth. Advancement to each higher level was signaled at the outset with written text on the screen. Each level contained three trials.

Participants were instructed to *mirror* the person in the video segments – that is, to use their right hand and arm when the left hand and arm were used in the video, and vice versa. This protocol was adopted because pilot research indicated that mirroring movements rather than producing direct copies led to greater success for most individuals. Additionally, participants were asked to begin each trial with their hands at their sides, and to return them to their sides after completing each movement. They were told that movements could be recalled in any order. Performance was videotaped and scored offline. However, an experimenter was present throughout the experiment and scored responses online as well, in order to encourage accuracy and adherence to instructions.

**Figure 1:**
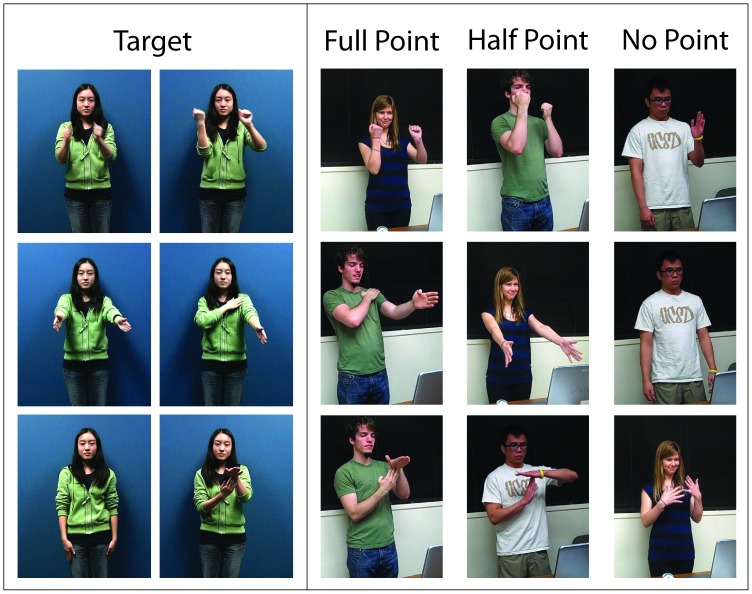
Recall of three target movements by three representative individuals. Participants were instructed to mirror the actress's movements; however, for clarity, her postures have been reversed in the freeze frames, enabling direct comparison between target and recall. All individuals have given written informed consent to publication of their image.

All correctly recalled movements were awarded one point (Figure 1). No penalty was imposed for movements reproduced with hesitation or slight deviations from the target (e.g. slightly bent elbows or curved fingers used to enact a target originally executed with straight arms and flat hands). Half points were awarded for responses that clearly reflected some recollection of the target movement, but were not entirely accurate. For instance, in the case of targets with wide ranges of motion, typically involving the arms and shoulders, a half point would be awarded to a reenactment in which all of the elemental movements were correctly performed except one (Figure 1, middle column of middle row). In the case of items with more limited motion, performed primarily in central space, a half point would be awarded if a participant correctly reproduced the target hand/arm configuration, but in the wrong location or with the wrong orientation (Figure 1, middle column of top and bottom rows), or in the case of configurations involving primarily the fingers, if a digit next to the target digit was used.

Importantly, to earn a half point, a reenactment was only allowed to deviate from the target along a single parameter. The only exception to this guideline was the case of asymmetrical movements that were reproduced as direct copies. In such instances, it was manifest that the participant had remembered the critical elements of the target. However, it was impossible to determine whether the execution of a direct copy was simply a performance error, or the result of inaccurate encoding or recall. For this reason, only a half rather than full point was awarded.

In keeping with scoring practices used in the Corsi block and sentence span tasks, items that were not reproduced accurately (i.e. substitutions or intrusions) or were not reproduced at all (omissions) were not awarded any points. Analysis of sixteen participants' performance revealed that omissions comprised the overwhelming majority of errors (72%), followed by intrusions (24%), and substitutions (4%). These statistics suggest that a primary limiting factor was decayed memory traces rather than confusion or proactive interference.

From each participant's performance, two measures were computed – a traditional, absolute span score based on the maximum number of elements that could be correctly recalled within a given level, as well as a total memory span score based on the total number of items recalled across all trials. There is some disagreement in the literature on memory span tasks regarding the merit of each measure (for review see[16]. One of the main criticisms of absolute span scoring centers around the discrete nature of this measure, which only encompasses five or six levels in the present study. Since this type of score is distributed along such a limited range, the detectable variance between individuals is reduced, and statistical power is potentially reduced as well. For this reason, some researchers prefer a continuous measure such as total memory span [16], [30]. On the other hand, traditional span measures offer a useful heuristic for grouping individuals according to a fairly simple, easily operationalized set of criteria. Validation for such measures can be found in the fact that a number of investigators have reported a high degree of correlation between continuous and discrete measures of memory span [31], [32], [33].

Total memory span on the movement span task was calculated simply by summing all of a participant's full and partial points across all trials. Absolute spans were determined according to the following system: for each consecutive level that an individual was able to score at least half of the total points possible within that level, his span score was incremented by one. Or in other words, the final span score was defined as one less than the first level at which he did not reach criterion. In cases where a participant achieved criterion on a subsequent level after his span had already been established, his final span was incremented by a half point. Notably, this method for calculating absolute span differs somewhat from approaches adopted in other WM span tasks (see below). At any given level, an individual could reach criterion either by recalling all of the items in some of the trials or recalling at least half of the items in all of the trials. For instance, at level 3, there are three trials with three items each, yielding nine items total. At a minimum, a person could reach criterion at this level by correctly recalling all three items on one trial, and at least 1.5 items on the other trials. Alternatively, he or she could recall 1.5 items on each trial. Thus, a span score of 3 reflects the set size at which an individual can correctly reproduce at least half of the items on each trial.

This approach to assessing span scores was chosen for two reasons. First, pilot data suggested that it yields a more even distribution of participants across the five levels than a more traditional scoring procedure. Secondly, because individual items may have varied in difficulty, it was deemed more appropriate to employ a procedure that took into account performance on all of the items within a level rather than only those items occurring on trials in which full recall was achieved.

To assess the stability of participants' performance over time, twelve individuals completed the movement span task in two separate sessions spaced approximately one week apart. Cronbach's alpha, computed between span scores obtained on each session, reflected a high level of score stability across sessions (absolute span scores: α = .94; total recall scores: α = .91).

To assess the stability of the scoring system across different raters, 82 movement span data sets were divided into three comparably sized groups and subjected to two separate evaluations. Correlation tests between the sets of ratings for each group revealed considerable agreement for both absolute span (r = 0.92, 0.85, 0.80) and total recall scores (r = 0.98, 0.90, 0.84) (All correlation coefficients were significant at a level of p<.0001). These outcomes indicate that with appropriate training, different raters are able to consistently assess participants' performance. The complete set of scores analyzed for this study was derived from the contributions of four experienced raters.

#### Corsi Block Task

The Corsi block-tapping task [34] is a widely used test of spatial skills and non-verbal WM. In the computerized variant implemented here, an asymmetric array of nine squares was presented on the monitor. On each trial, a subset of the squares would flash in sequence, though no square flashed more than once. Participants were instructed to reproduce each flash sequence immediately afterwards by clicking their mouse in the appropriate squares in the order that the flashes had occurred. Sequences ranged from four to nine flashes and were presented in blocks of five. Successfully reproducing at least one sequence in a block led to advancement to the next level. The task terminated when no sequences were correctly reproduced within a level or when level nine was completed. An individual's absolute span score was the highest level at which at least one sequence was correctly replicated [35]. Total memory span was assessed by summing all trials in which all items were correctly recalled in sequence.

#### Sentence Span Task

This assessment tool is based on Daneman and Carpenter's [36] pioneering work, which demonstrated robust correlations between an individual's span score and reading comprehension abilities. In the version utilized here, participants listened to unrelated sentences and remembered sentence final words. This protocol strongly encouraged rehearsal through subvocal articulation – presumably engaging the phonological loop proposed by Baddeley and colleagues to mediate verbal WM. Each trial concluded with a cue to write down the remembered words in any order. Trials contained between two and five sentences each, depending on the level, which increased systematically as the test progressed. Accordingly, trials were blocked by level, beginning with a block of level 2 trials, and ending with a block of level 5 trials. Filler trials with comprehension questions were included to encourage attention to the meaning of all sentences as participants held final words in memory through internal repetition.

An individual's absolute sentence span was the highest consecutive level at which all sentence final words were accurately recalled on at least two of the three trials in a block. In keeping with scoring procedures employed elsewhere [30], [31], [36], an additional half point was added in cases in which participants correctly completed at least two thirds of a later block after their span had already been established at a lower level. Total sentence span was the aggregate count of all correctly recalled words on non-filler trials [31].

#### Gesture Classification Task

**Figure 2:**
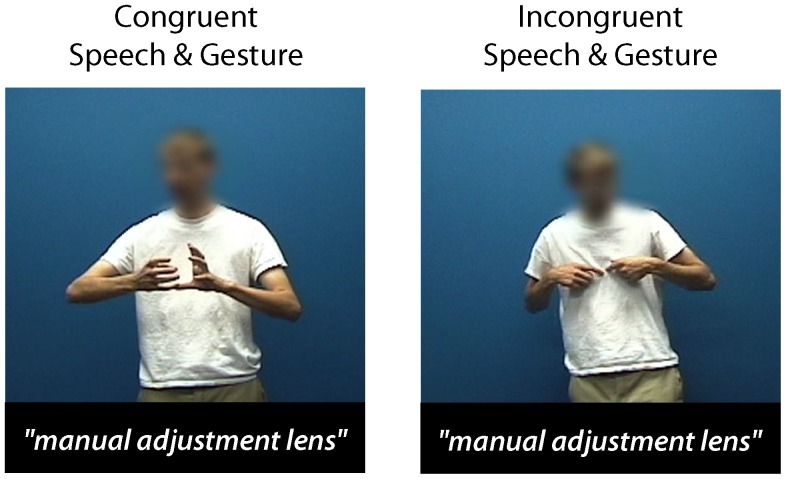
Still images taken from videos used in the gesture classification task. Participants viewed videos comprised of either congruent (left) and incongruent (right) speech and gesture and judged whether or not the gestures “went with” the accompanying speech.

Stimuli for this task were taken from a corpus of video-recorded discourse in which a naïve individual described everyday events and experiences to an off-camera interlocutor. Congruent trials were created by extracting short segments (2–8 s) of footage in which the speaker's utterances were accompanied by depictive gestures. For each congruent item, a counterpart incongruent trial was derived by swapping the audio and video portions of each film clip such that the semantic relationship between speech and gestures was minimized (Figure 2). In other words, the meaning of speech and gestures was easily integrated in congruent clips, and difficult to combine in incongruent ones. In the case of incongruent items, the disconnect between the speaker's orofacial movements and the spliced in audio speech track was obscured by blurring the speaker's face. For each congruent counterpart, a similar blurring procedure was undertaken in order to maintain visual consistency across the two stimulus types. Ten naïve individuals rated materials for the degree of correspondence between speech and gestures on a five point Likert scale (1  =  highly incongruent; 5  =  highly congruent). The average rating was 2.2 (SD = .7) for incongruent videos, and 3.8 (SD = .8) for congruent ones.

A pair of lists was created such that across lists, each video and speech file were presented once as a congruent and once as an incongruent item, but no videos or speech were repeated within a list. All stimuli were presented in the center of a computer monitor. Each trial began with a title designed to provide a contextual framework for interpreting the upcoming discourse. Participants were instructed to read the titles, and to watch and listen to each video. Once a video was completed, they were required to classify the speech and gestures that they had just seen as either congruent or incongruent by clicking with the mouse. To evaluate the internal consistency of this task, each participant's data set was divided in half, and Cronbach's alpha was calculated to compare accuracy of speech-gesture classification across the two segments. This analysis revealed high reliability between performance on the individual sets of items (α = .95), suggesting that the elements of the test consistently assessed a common underlying construct.

### Data Analysis

Estimates of d' – a measure of signal detection [37] – were obtained on the basis of hit and false alarm rates on the gesture classification task. (Measures of bias toward classifying items as either congruent or incongruent were not included in the analysis because bias toward either response type was not expected to be related to WM capacity.) To assess the relationship between this measure of sensitivity to the semantic aspects of body movement and working memory abilities, two multiple regression tests were conducted. The first modeled sensitivity to gesture meaning, as indexed by dprime estimates, using absolute span values obtained from the three WM tasks (movement span, Corsi block span, and sentence span). The second modeled the same response variable using total recall scores as predictor variables. All measures were standardized for comparison purposes.

## Results and Discussion

**Figure 3:**
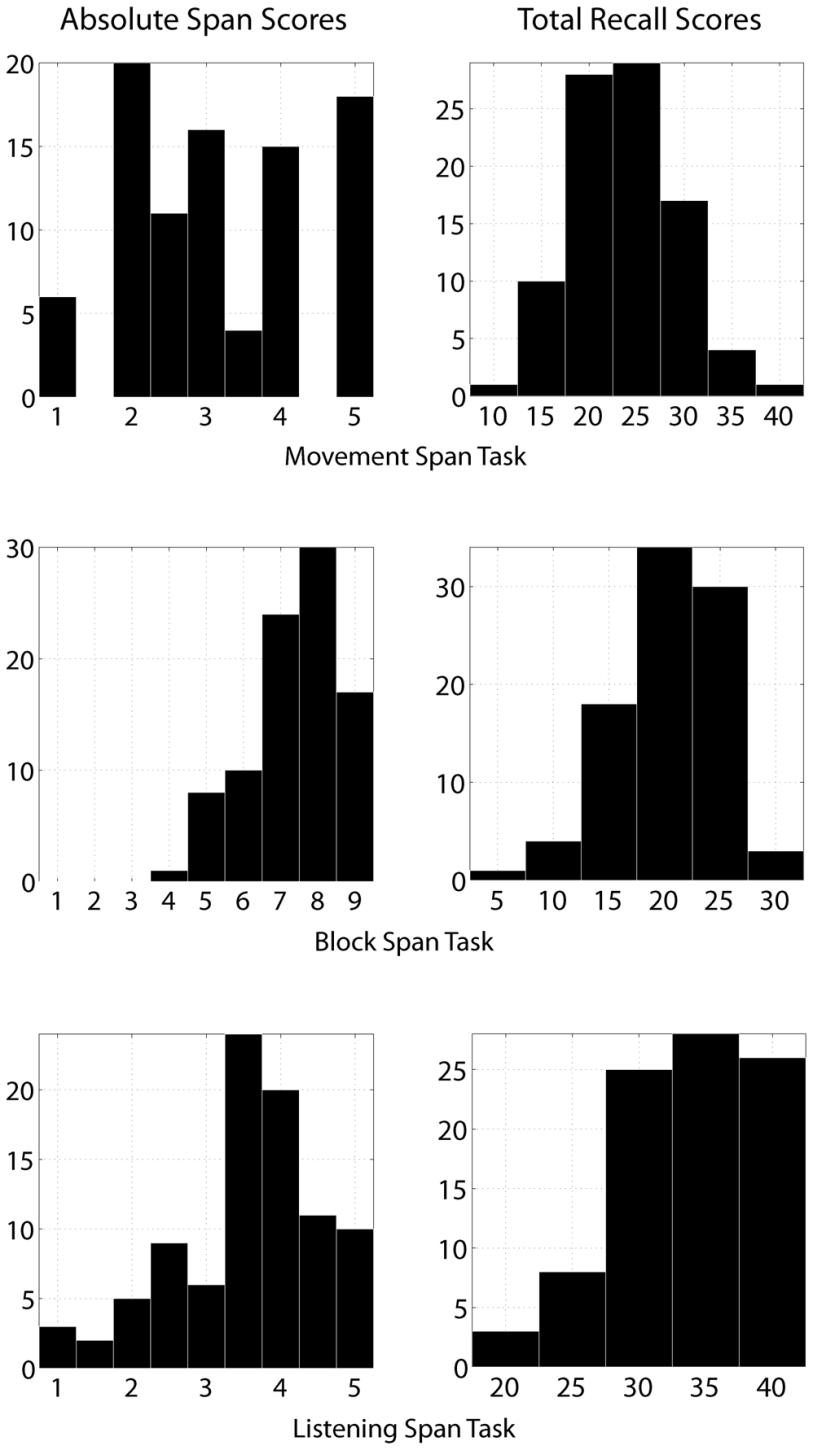
Histograms of absolute span and total recall scores. Note that mean recall scores reflect total items for the movement and sentence span tasks, whereas they reflect the number of correctly reproduced sequences for the Corsi block task.

Descriptive statistics and zero-order correlations of all scores are presented in Table 1. In the case of the Corsi block and the sentence span tasks, the majority of participants earned absolute span scores that were above the mid-point of the scale (see Figure 3), indicating robust WM abilities. On the other hand, the majority of absolute movement span scores tended to fall either above or below the mid-point (that is, 3), suggesting that at least some individuals who performed well on the Corsi block or sentence span tasks nevertheless performed poorly at reproducing meaningless movements.

**Table 1:** Zero-order correlations and descriptive statistics for scores on the span and gesture classification tasks.

	1	2	3	4	5	6	7
1. Gesture Sensitivity	―						
2. Movement Span	**0.38**	―					
3. Movement Recall	**0.45**	**0.80**	―				
4. Corsi Block Span	−0.01	0.13	0.22	―			
5. Corsi Recall	0.07	0.18	0.26	**0.82**	―		
6. Sentence Span	0.05	0.18	0.12	0.08	−0.00	―	
7. Word Recall	0.05	0.12	0.09	0.04	0.01	**0.78**	―
Mean Score (SD)	2.2 (0.9)	3 (1)	23.8 (5.7)	7 (1)	20.4 (4.6)	3.5 (1)	33.2 (5.8)
Range (Min, Max)	3.8, −0.4	1, 5	12.5, 38	4, 9	7, 29	1, 5	22, 41

Bolded font indicates values that survived Bonferroni correction (p<0.001).

It is noteworthy that for all three tasks, absolute span and total recall span scores were highly correlated. This finding echoes reports by other studies of verbal and visuo-spatial working memory [31], [32], [33], and suggests that depending on the research goals and questions, either measure may be used. Also consistent with prior research was the absence of any correlation between our measures of verbal (Sentence Span and Total Word Recall) and visuo-spatial (Corsi Block Span and Total Corsi Recall) working memory capacity, consistent with the claim that these tap dissociable components of working memory [38].

**Figure 4:**
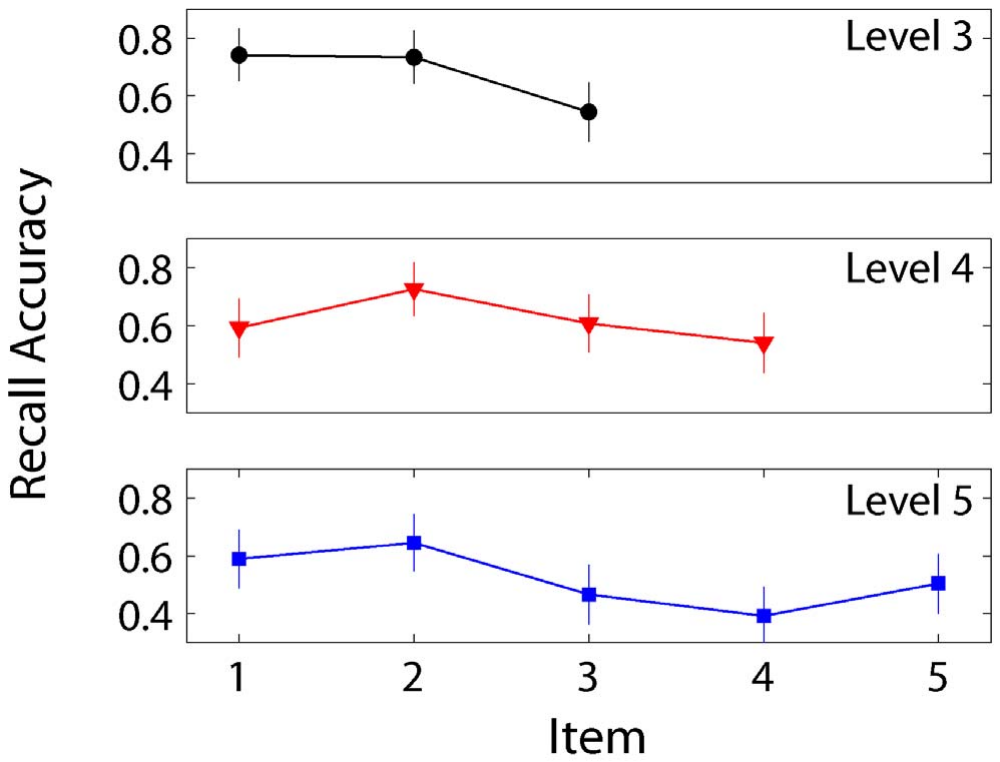
Mean recall accuracy for items at levels three through five with 95% confidence intervals. The y-axis reflects the proportion of participants who correctly reenacted the target movements at each position in the sequence.

The serial position curves for sequences with three, four, and five meaningless movements are plotted in Figure 4. For each participant, recall accuracy at each position was averaged across trials within a level and submitted to a single factor repeated measures ANOVA. For all three sequence lengths, main effects of serial position were found (level 3: F(2,89) = 19, p<.05; level 4: F(3,89) = 10, p<.05); level 5: F(4,89) = 19, p<.05). Follow-up t-tests revealed primacy effects at level three and four. At level five, both primacy and recency effects were observed.

Mean accuracy on the gesture classification task was 80% (S. D. 13%). Since *d'* is calculated by taking both correctly classified items as well as false alarms into account, it reflects participants' overall sensitivity to the semantic relationships between speech and gesture, as opposed to their accuracy on any one type of response. Values close to zero indicate poor discrimination of congruent versus incongruent videos, whereas larger *d'* scores occur as signal detection increases.

**Figure 5:**
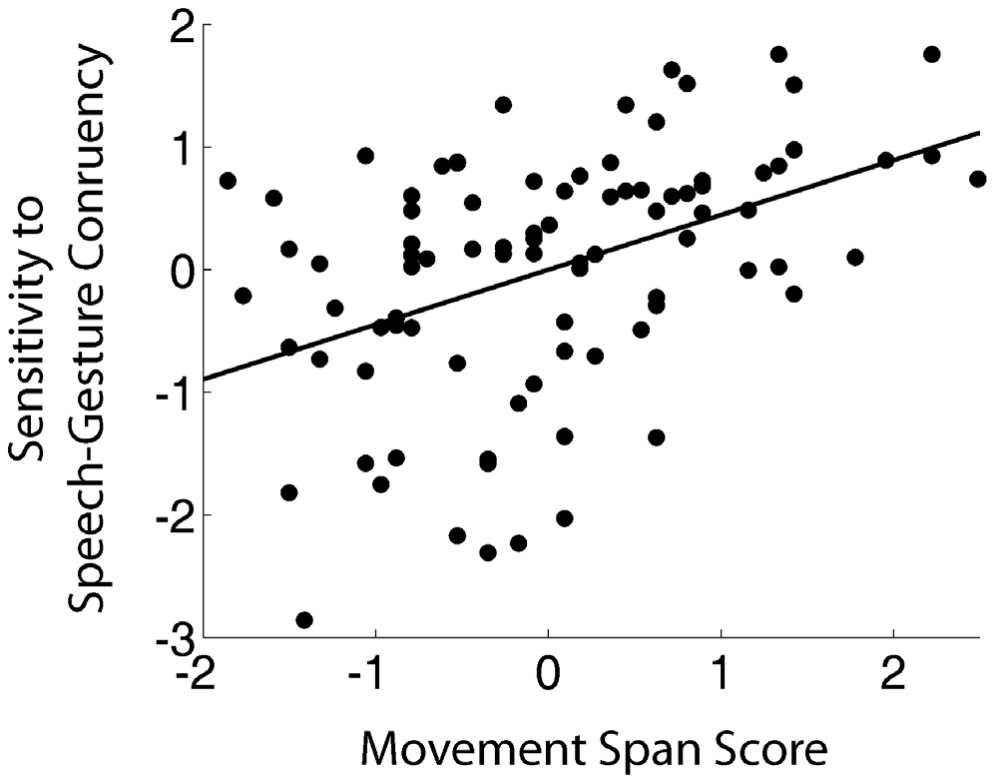
Zero-order correlation between d' on gesture classification task and movement span scores. Sensitivity to gesture meaning increased linearly with increased capacity to remember and reproduce body movements.

Figure 5 plots the zero-order correlation between d' and absolute movement span scores. A multiple regression test revealed that absolute movement span reliably predicted sensitivity to gesture meaning (β = 0.40, t = 4.0, p<0.05). That is, participants who were able to remember and reproduce greater quantities of movements were able to better distinguish gestures that matched a speaker's concurrent utterance from those that did not match. No consistent relationship was found between performance on the gesture classification task and the Corsi block (β = −0.06, t = −0.60, n.s.) or sentence span tasks (β = −0.01, t = −0.14, n.s).

A second multiple regression model using total recall scores as predictor variables yielded a similar pattern of results. The total number of accurately reproduced movements reliably predicted performance on the gesture sensitivity task (β = 0.46, t = 4.6, p<0.05), whereas the quantity of recalled block locations (β = −0.04, t = −0.46, n.s.) and sentence final words (total recall: β = 0.03, t = 0.32, n.s.) did not. These outcomes suggest that of the three span tasks tested here, only the movement span task predicted participants' accuracy in explicitly judging the presence of meaningful relationships between gestures and their accompanying speech.

This relationship between movement span scores and sensitivity to speech-gesture congruency is all the more intriguing given the proposal advanced by Rumiati and colleagues [5], [39] that meaningful and meaningless actions can engage distinct processing pathways. In their dual route model, meaningful actions can be imitated either through the activation of stored functional knowledge in semantic memory, or through direct visual analysis. On the other hand, meaningless actions can only be reproduced through the direct visual route. In the present study, it is likely that the movement span task recruited systems associated with the direct route, whereas the gesture classification task involved at least some semantic processing of the speakers' utterances and gestures. However, the positive relationship between d' and movement span scores suggests that visual analysis was also important for the gesture classification task.

This circumstance can be explained at least in part by the indeterminate semantic status of co-speech iconic gestures. Unlike language, these gestures do not convey meaning through entrenched symbolic mappings. Rather, they rely on perceptual similarity or shared relational features relative to the referents that they denote. Thus, it is not surprising that interpreting an iconic gesture would depend heavily not only on stored semantic knowledge, but also on the kinds of action processing implicated in the direct visual route proposed by Rumiati and colleagues and likely engaged during the movement span task.

In addition to the scoring procedure described above, we also subjected a subset of the data (81 participants) to a serial recall scoring procedure. On this scoring procedure, participants were not awarded points unless they reproduced the movements in the same order as the original video. Notably, this yielded a comparable pattern of results to those observed with our original (free recall) scoring procedure. On average, individuals recalled 21.3 (SD = 6) items and earned absolute span scores of 3 (SD = 1). A multiple regression test revealed a reliable predictive relationship between performance on the gesture classification task and both serial movement span scores (β = 0.32, t = 2.9, p<0.05), as well as serial total recall scores (β = 0.28, t = 2.5, p<0.05). These results are all the more surprising given the fact that participants were not asked to reproduce items in the same sequence that they were presented. Outcomes are consistent with Smyth and Pendleton's [4] report revealing that individuals recalled similar quantities of items and were impacted by different modalities of interference in similar ways when their performance was evaluated through both serial and free recall procedures. In the present case, it appears that many participants spontaneously employed serial recall strategies even in the absence of explicit instructions to do so.

As implemented here, the movement span task appears to reflect aspects of immediate memory that are important for retaining body-specific visual input. Notably, zero-order correlations revealed no reliable link between verbal WM and performance on the movement span task. While it is certainly conceivable that verbal encoding strategies could have been used during the task (e.g. rehearsing names of body parts or other movement parameters specific to the items in a trial), it appears that such strategies were either not employed or were unhelpful.

Visuo-spatial WM abilities were also unrelated to participants' movement span scores. This outcome is consistent with research suggesting the dissociability of memory for body configurations and spatial locations [6]. For example, movements to spatial targets have been shown to interfere with memory for spatial locations, whereas patterned, body-centered movements (e.g. tapping the head and hips) interfere with memory for various body postures (e.g. extending arm across body and touching opposite shoulder) [4], [7], [8]. However, we do not entirely rule out the possibility that immediate memory for locations in space may have contributed to performance on the movement span task, as the test items can be construed as involving both spatial and kinesthetic elements. Because all body movements are intrinsically produced in space, participants needed to remember not only novel configurations of the hands and arms, but also certain spatial parameters, such as orientation to the right, left, or center of the body, upward versus downward trajectories, and so forth.

The scoring scheme used here was designed to compensate somewhat for the complex relationship between space and body movement by awarding half points when participants did not accurately reproduce a certain spatial feature of an item, but clearly remembered the main components of the target configuration. It is also possible that employing a task that emphasizes motoric rather than visual encoding of movements would diminish possible contributions of visuo-spatial WM to performance on the movement span task. For example, participants could be instructed to rehearse each item on their own bodies during the study phase.

This proposal is consistent with research suggesting a strong link between spatial rehearsal, spatial attention, and oculomotor control [40], [41], [42]. In fact, it has been proposed that the rehearsal component of WM can be reduced in essence to sustained preparation to execute some form of voluntary action (ocular, manual, verbal, and so forth) [40]. In this view, some overlap in systems mediating memory for spatial locations, on the one hand, and body configurations to be realized in peripersonal space, on the other, is warranted.

A drawback of the present study is that time constraints precluded evaluation of WM abilities through multiple measures. Because null effects must be interpreted with caution, the claim that sensitivity to speech-gesture congruency is not reliably linked to visuo-spatial or verbal WM abilities would certainly be bolstered if comparable patterns of results were obtained from two or more tests designed to tap these modalities. An additional consideration for future research involving the movement span task concerns the presentation order of trials. In order to minimize the impact of strategies that may become viable when participants are able to anticipate the number of items to be presented on each upcoming trial, it could prove useful to intermix trials from different levels in a randomized fashion [43].

## Conclusions

The movement span task assesses individuals' ability to remember and reproduce meaningless configurations of the body. During the encoding phase of a trial, participants watch short videos of meaningless movements presented in sets varying in size from one to five items. Immediately after encoding, they are prompted to reenact as many items as possible. Performance on this task was not correlated with measures of verbal or visuo-spatial WM. Perhaps most impressively, however, performance on the movement span task did predict performance on a separate test that relied heavily on body-centered processing – classification of a speaker's gestures as either congruent or incongruent with his accompanying speech. Notably, participants' success on the gesture classification task was linked *only* to their ability to remember and reproduce body configurations. Tests designed to assess visuo-spatial (reproducing a sequence of spatial locations) or verbal (remembering sentence final words) WM were not predictive of sensitivity to gesture-speech congruity. These data suggest that the movement span task is particularly well-suited for assessing the contribution of WM to individual differences in tasks that fundamentally involve body based processing, and may account for variance that might otherwise remain unexplained.
